# The Regulatory Effects of Exercise and Metformin on Biomarkers in Obesity: A Focus on Uric Acid, Irisin, Adiponutrin, Adropin, and Copeptin

**DOI:** 10.3390/medicina61030399

**Published:** 2025-02-25

**Authors:** Taner Akbulut, Vedat Cinar, Emsal Cagla Avcu, Yavuz Yasul, İsa Aydemir, Tuncay Kuloglu, Gokhan Artas, Suleyman Aydin

**Affiliations:** 1 Department of Coaching Education, Faculty of Sports Sciences, Firat University, Elazig 23119, Turkey; 2 Department of Physical Education and Sports Teaching, Faculty of Sport Sciences, Firat University, Elazig 23119, Turkey; cinarvedat@hotmail.com; 3 Department of Coaching Education, Faculty of Sport Sciences, Sivas Cumhuriyet University, Sivas 58140, Turkey; emsalcagla.avcu@hotmail.com; 4 Property Protection and Security Division, Bafra Vocational School, Ondokuz Mayıs University, Samsun 55400, Turkey; yavuz.yasul@omu.edu.tr; 5 Faculty of Education, Physical Education and Sports, Hakkari University, Hakkari 30000, Turkey; aydemirisa23@gmail.com; 6 Department of Histology and Embryology, Faculty of Medicine, Firat University, Elazig 23119, Turkey; tkuloglu@firat.edu.tr; 7 Department of Medical Pathology, Faculty of Medicine, Firat University, Elazig 23119, Turkey; gartas@firat.edu.tr; 8 Department of Biochemistry, Faculty of Medicine, Firat University, Elazig 23119, Turkey; saydin1@firat.edu.tr

**Keywords:** exercise, metformin, obesity, adipokines, myokines

## Abstract

*Background and Objectives*: Obesity has become one of the most significant health problems nowadays, with its prevalence rapidly increasing. Approaches such as diet and exercise play an important role in the treatment of obesity. This study aimed to investigate the responses of uric acid, irisin, adiponutrin, adropin, and copeptin levels to exercise and metformin intervention in obesity. *Materials and Methods*: Thirty-six male Sprague–Dawley rats were randomly divided into seven groups: healthy control (HC), sham (S), obese control (OC), metformin (M), exercise (E), metformin + exercise (ME), and decapitation (D). After obesity was induced through a 12-week high-fat diet, obese rats underwent a 4-week aerobic exercise and metformin intervention. *Results*: Uric acid, irisin, adiponutrin, adropin, and copeptin levels were determined using an ELISA method. Copeptin levels significantly decreased in the ME group (*p* < 0.001). Irisin levels significantly increased in the E and ME groups (*p* < 0.001). The most notable increases in adropin levels occurred in the E and ME groups (*p* < 0.001). Uric acid levels were highest in the OC group but significantly lower in the E and M groups (*p* < 0.001). Adiponutrin levels did not change in response to exercise or metformin intervention in obesity (*p* > 0.05). *Conclusions*: These findings suggest that exercise and metformin intervention may play an effective role in obesity management.

## 1. Introduction

Obesity is defined as a complex and multifactorial disease characterized by excessive fat accumulation in the body, leading to negative impacts on health [1]. According to the World Health Organization, a body mass index (BMI) over 25 is classified as overweight, while a BMI over 30 is considered obese [2]. Today, overweight and obesity have become issues that seriously threaten public health and have reached epidemic proportions [3]. Unhealthy dietary habits, chronic positive energy balance, reduced physical activity, and a sedentary lifestyle play a significant role in the development of obesity. These factors lead to fat accumulation and structural changes in adipose tissue, negatively affecting metabolic processes and contributing to the development of obesity [4].

The primary treatment approaches for obesity include diet interventions supported by exercise and cognitive behavioral therapy. These methods can contribute to sustainable weight loss [5]. However, traditional approaches aimed at changing dietary habits and increasing physical activity do not always lead to lasting results. In many individuals, after long-term diet and exercise programs, a regain of fat mass and body weight is common. Moreover, bariatric surgery, which can be an effective alternative, is not viable in all cases [6]. Although lifestyle interventions form the foundation of obesity treatment, pharmacotherapy becomes necessary in many cases. In this context, various systematic reviews on the use of metformin in obesity treatment provide valuable information on its effects on weight management [7,8,9,10]. Current evidence suggests that the effect of metformin on weight change may be more related to reductions in calorie intake rather than to an increase in energy expenditure. However, the use of metformin as a primary treatment for obesity or as an effective weight-loss agent has not yet been fully established [9].

Obesity significantly affects the expression levels and regulatory mechanisms of molecules that play a critical role in maintaining energy homeostasis. Irisin is a hormone with anti-obesity and anti-diabetic properties that regulates adipose tissue metabolism and glucose homeostasis by converting white adipose tissue to brown adipose tissue [11]. Research has shown that irisin plays an important role as both a protective measure and a biomarker for comorbidities associated with obesity and cardiometabolic disorders [12]. Adiponutrin is an adipocyte protein regulated by energy balance, and its mRNA levels have been shown to increase in obese Zucker rats [13]. Adropin is a hormone that regulates energy homeostasis and significantly affects lipid and glucose metabolism, with its levels reported to decrease in obesity [14]. Copeptin is a part of the precursor protein of arginine vasopressin (AVP), which regulates fluid homeostasis and the endocrine stress response and has been associated with metabolic diseases [15,16]. Additionally, it has been shown that mature adipocytes and adipose tissue secrete uric acid, leading to increased uric acid levels in obesity [17]. Although some studies have investigated the connections between these molecules and obesity, it is clear that more research is needed on the effects of exercise and metformin interventions on these molecules in obesity. In particular, the responses of biomolecules such as adiponutrin and copeptin to exercise and metformin interventions in obesity have, to the best of our knowledge, not been examined before. Therefore, this study aims to provide new and significant insights into the responses of these biomolecules to interventions for obesity. Metformin, an effective agent in the treatment of obesity, regulates energy homeostasis through adenosine monophosphate-activated protein kinase (AMPK) activation, affects the metabolism of fatty acids, and suppresses adipogenesis, while exercise promotes the conversion of white adipose tissue to brown adipose tissue, increases energy expenditure, and supports the body’s fat burning process by increasing the oxidation of fatty acids [3,10]. Both interventions have the potential to influence these biological processes and changes at the molecular level. The main hypothesis of this research is that the levels of irisin, adiponutrin, adropin, copeptin, and uric acid will change with exercise and metformin interventions. In this context, the aim of the study is to examine the effects of exercise and metformin interventions on the levels of uric acid, irisin, adropin, adiponutrin, and copeptin in obese rats.

## 2. Materials and Methods

The study was conducted following an experimental research design with a posttest control group. First, ethics committee permission was obtained for the study from the Animal Experiments Local Ethics Committee at Firat University, Turkey, dated 5 May 2017 with decision number 2017/09 and decision number 120. The feed of the animals without obesity was obtained from the Experimental Animal Research Center at Firat University as normal rat feed (in pellets). This study adhered to the criteria of the National Institutes of Health for the Care and Use of Laboratory Animals. Experimental animals were kept in an environment with 12 h of darkness and 12 h of light at a standard room temperature (21 ± 1 °C). During the maintenance period, cages were cleaned regularly, and food, water, and cage interiors were checked daily. The study lasted a total of 17 weeks, comprising a 12-week obesity induction protocol, a 1-week adaptation exercise protocol, and a 4-week intervention protocol (exercise and metformin).

### 2.1. Research Groups and Termination

The sample size for the study was determined using G*Power software (3.1), which indicated that a minimum of five rats per group would be required to detect a statistically significant difference at a 0.05 significance level (alpha), 0.8 statistical power (1-beta), and an effect size of 1.81 under a two-tailed alternative hypothesis (H1). In total, 36 Sprague–Dawley rats were used in the study. These rats were divided into seven distinct groups using simple randomization: healthy control (HC), sham (S), decapitation (D), obese control (OC), metformin (M), exercise (E), and exercise + metformin (ME).

Healthy control group (HC; n = 5): The rats were fed a standard diet (rat pellet food) throughout the study and were not subjected to any interventions.

Sham group (S; n = 5): Obesity was induced with a high-fat diet for 12 weeks, and then for 4 weeks, 150 mg/kg of water was administered daily via gavage.

Obese control group (OC; n = 6): Obesity was induced with a high-fat diet for 12 weeks, and no additional interventions were applied.

Metformin group (M; n = 5): Obesity was induced with a high-fat diet for 12 weeks, and then for 4 weeks, 150 mg/kg of metformin was administered daily via gavage.

Exercise group (E; n = 5): Obesity was induced with a high-fat diet for 12 weeks, and then for 4 weeks, treadmill exercise was performed 5 days per week for 30 min per day at a 20 m/min speed and 0° incline.

Metformin + exercise group (ME; n = 5): Obesity was induced with a high-fat diet for 12 weeks, and then for 4 weeks, 150 mg/kg of metformin was administered daily via gavage, along with treadmill exercise 5 days per week for 30 min per day at a 20 m/min speed and 0° incline.

Decapitation group (D; n = 5): The D group was sacrificed before the start of the experimental process.

**Figure 1:**
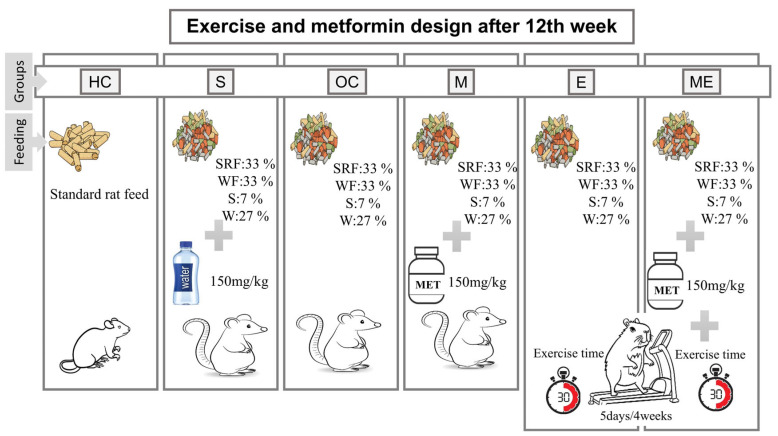
Experimental research design details of diet, exercise, and metformin intervention. HC: healthy control group, S: sham group, OC: obese control group, M: metformin group, E: exercise group, ME: metformin + exercise group, SRF: standard rat feed, WF: whole-fat sweetened milk, S: sucrose, W: water.

At the end of the study, twenty-four hours after the final exercise and metformin intervention sessions, all rats were decapitated after being fasted overnight, and blood samples were collected. Blood samples were collected in EDTA tubes containing aprotinin and centrifuged at 4000 rpm for 5 min (Hettich, Kirchlengern, Germany). Plasma levels of uric acid, irisin, adropin, copeptin, and adiponutrin were analyzed using the ELISA method, following the protocol provided in the kit catalog. The whole experimental design is shown in Figure 1.

### 2.2. Exercise Protocol

The rats in the exercise groups (E and ME) spent the first week on the treadmill (KN-73; Natsume Seisakusho Co., Ltd., Tokyo, Japan) performing an adaptation exercise protocol (5 days a week, 30 min a day, 20 m/min speed, and 0° incline). After this phase, they underwent a treadmill exercise protocol for 4 weeks (5 days a week, 30 min a day, 20 m/min speed, and 0° incline). All exercise sessions were conducted between 10:00 AM and 12:00 PM.

### 2.3. Diet Protocol and Determining Obesity

The D group was sacrificed prior to the initiation of the experimental procedures. The CH group was fed a standard rat diet and did not undergo any interventions. The S group, OC group, M group, E group, and ME group were subjected to a diet-induced obesity (DIO) protocol for 12 weeks. During this period, the rats were fed ad libitum with a diet comprising 33% rat chow, 33% whole-fat sweetened milk, 7% sucrose, and 27% water.

After 12 weeks, the rats’ obesity status was assessed by calculating their body mass index (BMI) using the formula BMI = [weight (g)/height (cm)^2^]. Height was determined by measuring the distance from the tip of the nose to the anus. Rats with a BMI ≥ 1.00 g/cm^2^ were classified as obese. By the end of the 12-week period, obesity induction was complete.

### 2.4. Metformin Intervention

Metformin supplementation was exclusively provided to the M and ME groups as part of the experimental protocol. The administration of metformin commenced following the induction of obesity in the rats and was synchronized with the initiation of the exercise intervention. Supplementation was carried out using the gavage method, ensuring precise dosage delivery. Each rat received metformin at a standardized dose of 150 mg/kg/day over a four-week period, administered in conjunction with a normal diet to evaluate its combined effects with exercise and dietary interventions [18]. Metformin was administered 30 min before exercise.

### 2.5. Biochemical Analyses

ELISA kits were employed for biochemical analyses, including those for uric acid (201-11-4529; Sunred Biological Technology Co., Ltd., Shanghai, China), irisin (201-11-1713; Sunred Biological Technology Co., Ltd., Shanghai, China), adropin (201-11-3361; Sunred Biological Technology Co., Ltd., Shanghai, China), copeptin (SRB-T-88867; Sunred Biological Technology Co., Ltd., Shanghai, China), and adiponutrin (201-11-1768; Sunred Biological Technology Co., Ltd., Shanghai, China). The Bio-Tek ELX50 microplate washer (BioTek Instruments, Winooski, VT, USA) was utilized for automatic plate washing, while absorbance values at 450 nm were measured using the ChroMate Microplate Reader P4300 (Awareness Technology Instruments, Palm City, FL, USA). The experimental validity of the ELISA kits was ensured according to the protocol outlined by Aydin [19,20].

### 2.6. Statistical Analysis

The data were presented as mean and standard deviation. The effect size of significant differences was presented using the formula for Hedge’s g, which is defined as $=\frac{M1-M2}{SDpooled}$ [21]. Body mass and BMI changes in different groups (G) and weeks (W) and their interaction (G × W) were analyzed by repeated measures ANOVA followed by Dunnett’s T3 test [22]. Differences in the levels of uric acid, irisin, adropin, copeptin, and adiponutrin between the groups were analyzed using the Brown–Forsythe ANOVA test followed by Dunnett’s T3 test [23,24]. In addition, the Pearson Correlation test was used to determine the relationship between variables. All analyses were performed using the SPSS 22.0 software package. Statistical significance was determined at the 0.05 level.

## 3. Results

Table 1 demonstrates that when analyzing the effects of exercise and metformin on body mass, different levels of significance were found for time, interventions, and their interactions. The three factors were found to be affected by inter-wk, intra-wk (W), and by the interaction (Wk × G) at a level of significance of *p* < 0.05, *p* < 0.01, and *p* < 0.001. Body mass at baseline, which was 234.7 g as the general mean, increased to 615.6 g at week 12 and decreased to 577.7 g after exercise and metformin administration. When only the group effects were evaluated, it was found that the body mass factor increased significantly in the S, O, E, M, and ME groups treated with DIO compared to that in the HC group at the 12th week (x^2^ = 17.003, *p* < 0.01). While this increase continued in the S and OC groups at the 17th week (x^2^ = 26.626, *p* < 0.001), a significant decrease was detected in the E, M, and ME groups in which exercise and metformin intervention were performed. In this week, the body mass level decreased the most in the ME group after the HC group.

**Figure 2:**
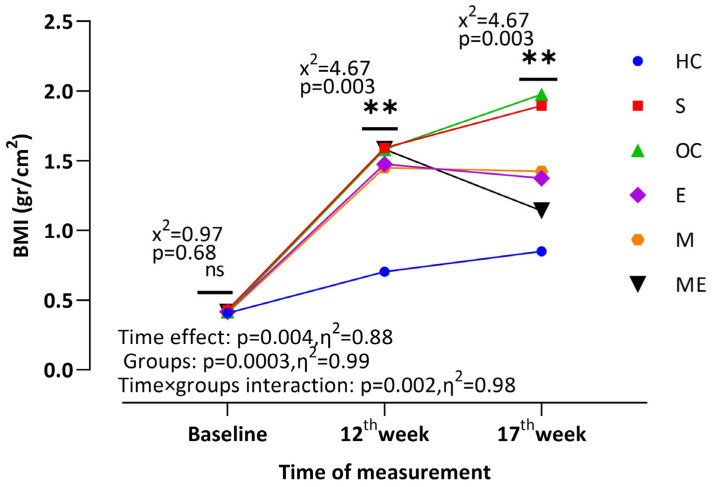
BMI characteristics of the groups. HC: control healthy group, S: sham group, OC: obese control group, M: metformin group, E: exercise group, ME: metformin + exercise group; x^2^ (inter-wk effect): BMI differences between weeks were analyzed by the Friedman test; Z (intra-wk effect): BMI differences within weeks were analyzed by the Wilcoxon test; x^2^ (Wk × G interaction): BMI differences between the groups were analyzed by the Kruskal–Wallis test, followed by the Mann–Whitney U test to compare two groups. **: *p* < 0.01.

Figure 2 demonstrates that the three factors were significantly affected by the inter-wk effect (*p* < 0.01), the intra-wk effect (*p* < 0.001), and their interaction (Wk × G: *p* < 0.01). The baseline BMI of the groups was 0.71, increasing to 2.32 at the 12th week and subsequently decreasing to 2.15 at the 17th week following the administration of both exercise and metformin. When only the groups were evaluated, the BMI level was 2.48 in the S group, 2.52 in the OC group, 2.45 in the E group, 2.47 in the M group, and 2.54 in the ME group in comparison to 1.45 in the HC group at the 12th week. This increase peaked at week 17, with a BMI of 2.69 in the S group and 2.74 in the OC group. However, after exercise and metformin administration, the BMI level decreased to 2.04 in the E group, 2.07 in M group, and 1.81 in ME group. In addition, the lowest BMI level at week 17th was found in the ME group after the HC group (1.53).

**Figure 3:**
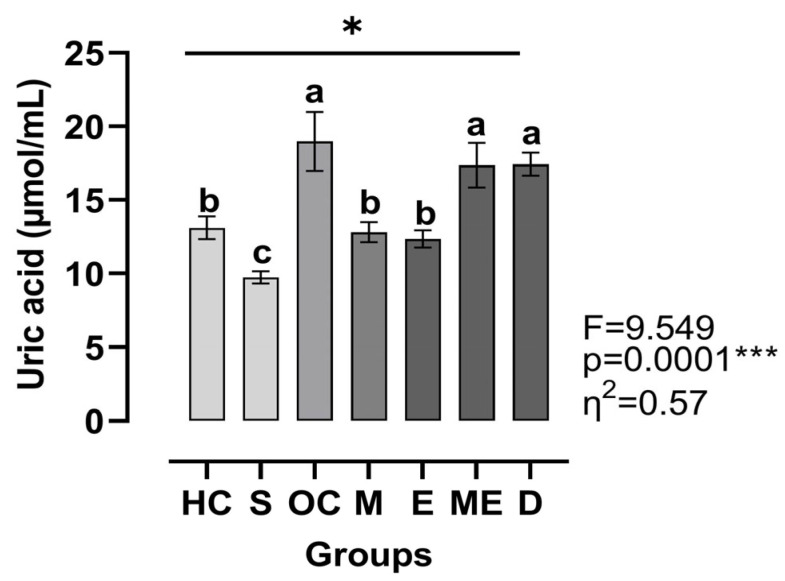
Plasma levels of uric acid. HC: healthy control group, S: sham group, OC: obese control group, M: metformin group, E: exercise group, ME: metformin + exercise group, D: decapitation group. Top line with *: different uric acid levels between the groups were analyzed by the Brown–Forsythe ANOVA test followed by Dunnett’s T3 multiple comparisons test; a, b, and c: dissimilar letters indicate significant differences, ***: *p* < 0.001.

Figure 3 shows that the uric acid levels were significantly affected between groups (η^2^ = 0.57, *p* < 0.001). The HC group was significantly different compared to the S group (Hedge’s g = 1.50, *p* < 0.05), OC group (Hedge’s g = 1.98, *p* < 0.05), ME group (Hedge’s g = 1.57, *p* < 0.05), and D group (Hedge’s g = 1.84, *p* < 0.05). Similarly, the S group was significantly different compared to the OC group (Hedge’s g = 3.90, *p* < 0.05), M group (Hedge’s g = 2.24, *p* < 0.05), E group (Hedge’s g = 1.97, *p* < 0.05), ME group (Hedge’s g = 3.89, *p* < 0.05), and D group (Hedge’s g = 5.03, *p* < 0.001. Notably, the S group displayed the lowest uric acid level. The OC group showed significant differences when compared to the M group (Hedge’s g = 1.02, *p* < 0.05) and E group (Hedge’s g = 1.10, *p* < 0.05) and had the highest uric acid level among the groups. Furthermore, significant differences were found between the M group and the ME group (Hedge’s g = 1.83, *p* < 0.05) and D group (Hedge’s g = 2.21, *p* < 0.05), as well as between the E group and the ME group (Hedge’s g = 2.18, *p* < 0.05) and D group (Hedge’s g = 2.71, *p* < 0.05).

**Figure 4:**
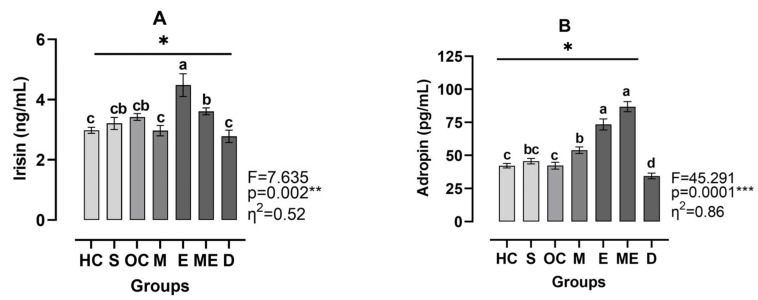
Plasma levels of irisin (**A**) and adropin (**B**). HC: healthy control group, S: sham group, OC: obese control group, M: metformin group, E: exercise group, ME: metformin + exercise group, D: decapitated group. Top line with *: different irisin and adropin levels between the groups were analyzed by the Brown–Forsythe ANOVA test followed by Dunnett’s T3 multiple comparisons test; a, b, c, and d: dissimilar letters indicate significant differences, **: *p* < 0.01, ***: *p* < 0.001.

Figure 4A shows that irisin levels were significantly different between groups (η^2^ = 0.52, *p* < 0.01). The E group exhibited the highest irisin levels, differing significantly from the HC group (Hedge’s g = 1.26), S group (Hedge’s g = 0.52), OC group (Hedge’s g = 0.82), M group (Hedge’s g = 0.01), and D group (Hedge’s g = 0.27) (all *p* < 0.05). According to Figure 4B, adropin level also varied significantly between groups (η^2^ = 0.86, *p* < 0.001). The ME group had the highest irisin level, significantly differing from the HC group (Hedge’s g = 1.26, *p* < 0.001), S group (Hedge’s g = 0.15, *p* < 0.001), OC group (Hedge’s g = 0.12, *p* < 0.001), M group (Hedge’s g = 0.09, *p* < 0.01), and D group (Hedge’s g = 0.08, *p* < 0.001). The E group had the second highest irisin level, significantly differing from the HC group (Hedge’s g = 0.14, *p* < 0.01), S group (Hedge’s g = 0.10, *p* < 0.01), OC group (Hedge’s g = 0.08, *p* < 0.01), M group (Hedge’s g = 0.06, *p* < 0.01), and D group (Hedge’s g = 0.13, *p* < 0.001). Furthermore the HC group, the S group, and the OC group were assigned to the same group, while group D exhibited the lowest levels of adropin.

**Figure 5:**
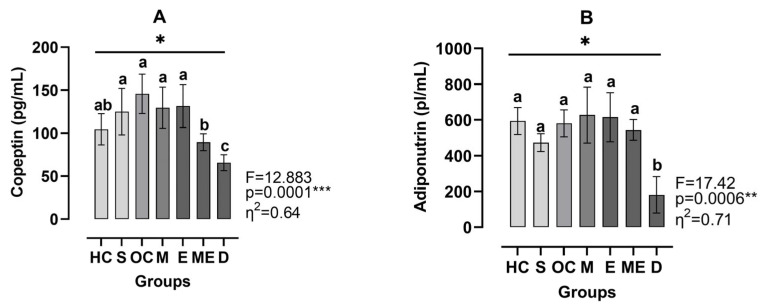
Plasma levels of copeptin (**A**) and adiponutrin (**B**). HC: healthy control group, S: sham group, OC: obese control group, M: metformin group, E: exercise group, ME: metformin + exercise group, D: decapitated group. Top line with *: different copeptin and adiponutrin levels between the groups were analyzed by the Brown–Forsythe ANOVA test followed by Dunnett’s T3 multiple comparisons test; a, b, and c: dissimilar letters indicate significant differences, **: *p* < 0.01, ***: *p* < 0.001.

Figure 5A shows that copeptin levels were significantly different between groups (η^2^ = 0.64, *p* < 0.001). The D group exhibited the lowest copeptin levels, differing significantly from the HC group (Hedge’s g = 0. 19, *p* < 0.05), S group (Hedge’s g = 0.013, *p* < 0.05), OC group (Hedge’s g = 0.24, *p* < 0.001), M group (Hedge’s g = 0.18, *p* < 0.01), ME group (Hedge’s g = 0.18, *p* < 0.01), and E group (Hedge’s g = 0.17, *p* < 0.01). Additionally, the ME group differed significantly from OC group (Hedge’s g = 0.17, *p* < 0.01), M group (Hedge’s g = 0.11, *p* < 0.05), and E group (Hedge’s g = 0.37, *p* < 0.05). According to Figure 5B, adiponutrin levels also varied significantly between groups (η^2^ = 0.71, *p* < 0.001). The D group exhibited the lowest adiponutrin levels, differing significantly from the OC group (Hedge’s g = 0. 20, *p* < 0.001).

**Figure 6:**
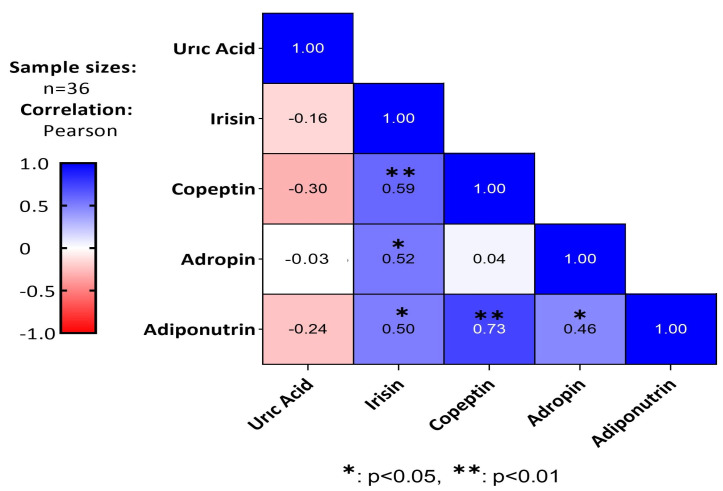
Heat map illustrating the correlations among uric acid, irisin, copeptin, adropin, and adiponutrin. The Pearson correlation coefficients (*r*) and corresponding *p*-values are presented for each pairwise relationship. Statistical significance is denoted as * *p* < 0.05 and ** *p* < 0.01.

According to Figure 6, there was a moderate positive and statistically significant correlation between adiponutrin and irisin (*p* < 0.05, *r* = 0.50), a strong positive and statistically significant correlation with copeptin (*p* < 0.01, *r* = 0.73), and a moderate positive and statistically significant correlation with adropin (*p* < 0.05, *r* = 0.46). Additionally, irisin showed a moderate positive and statistically significant correlation with adropin (*p* < 0.05, *r* = 0.52) and with copeptin (*p* < 0.01, *r* = 0.59) (Figure 6).

## 4. Discussion

The effects of a 4-week aerobic exercise and metformin intervention on plasma uric acid, irisin, adiponutrin, adropin, and copeptin levels in obese rats were investigated. The findings of the study revealed not only metabolic changes caused by obesity but also how exercise, metformin, and their combination affected biochemical parameters. To the best of our knowledge, this is the first study to demonstrate the response of adiponutrin and copeptin to exercise in obesity and their role in metformin efficacy. The main findings of the study were as follows: Adiponutrin levels did not change in response to exercise or metformin intervention in obesity, but copeptin levels significantly decreased in the metformin + exercise group. The most notable increase in irisin levels was observed in the exercise group, while the most significant rise in adropin levels occurred in the exercise and metformin + exercise groups. Additionally, uric acid levels were highest in the obesity group, but exercise and metformin interventions significantly reduced these levels. The findings of our research largely supported the validity of our hypothesis. However, the limited changes observed in certain parameters, such as adiponutrin, emphasize the need for further investigation into the mechanisms underlying the responses of these biomarkers to interventions.

Although plasma uric acid levels were found to be highest in the obesity, metformin + exercise, and decapitated groups compared to the other groups, no significant difference was observed among these three groups. However, the most notable increase was observed in the obesity group, which may indicate metabolic stress. Obesity can lead to an increase in uric acid synthesis and a reduction in its excretion, resulting in hyperuricemia [25]. Previous studies have reported a strong relationship between high uric acid levels and obesity [26]. A study conducted on overweight and obese patients found that elevated serum uric acid levels were positively associated with obesity [27]. In another study, serum uric acid levels were higher in the visceral obesity group compared to those in the lean group [28]. Furthermore, in the present study, a significant reduction in uric acid levels was observed in the metformin and exercise groups compared to those in the obesity group. The literature suggests that both metformin and exercise can reduce uric acid levels [29,30,31], and the results of the present study support these findings. This suggests that metformin and exercise may have the potential to reduce the risk of hyperuricemia associated with obesity. However, while a decrease in uric acid levels was observed in the exercise group and metformin group in separate applications, the fact that the metformin + exercise combination was not effective in reducing uric acid levels suggests that the mechanisms of action of these two interventions act differently when combined. Further research is warranted to explore the underlying mechanisms of this interaction.

Plasma adropin levels were found to significantly increase in the metformin, exercise, and metformin + exercise groups compared to those in the other groups. The increase in adropin levels with metformin and exercise interventions may be associated with the critical role of this peptide in regulating energy metabolism [32,33]. Similar findings regarding the effects of metformin and exercise on adropin levels have been reported in the literature. Zhang et al. demonstrated that 12 weeks of aerobic exercise increased serum adropin concentrations in obese adolescents [34]. Aydın et al. reported that 4 weeks of exercise elevated adropin levels in a metabolic syndrome model [35]. Additionally, Ticinovic-Kurir et al. showed that metformin and liraglutide treatment significantly increased adropin levels in obese male patients with type 2 diabetes [36]. These findings support the regulatory effects of exercise and metformin interventions on adropin levels.

According to our findings, exercise and metformin interventions in obesity did not cause a significant change in plasma adiponutrin levels. Metformin reduces fat accumulation, positively affecting the energy metabolism of adipocytes and suppressing lipogenesis [37,38]. This may explain the lack of significant change in adiponutrin levels, as adiponutrin is a protein involved in regulating energy balance and plays a role in lipogenesis rather than lipolysis [39]. Thus, the effect of metformin and exercise interventions may be based on the role of adiponutrin in relation to energy balance and lipogenesis. Additionally, the fact that low- to moderate-intensity and prolonged exercises largely meet the energy needs of skeletal muscles through fatty acid oxidation [40] could have contributed to the lack of change in adiponutrin levels. When fatty acid oxidation is prioritized during exercise, an increase in the levels of lipogenesis-related proteins, such as adiponutrin, may not be expected.

Research has emphasized that elevated copeptin levels are associated with obesity and metabolic diseases [16,41,42,43]. Our findings support this and showed that copeptin levels were elevated obesity groups compared to the healthy control group. However, exercise and metformin interventions in obesity did not affect copeptin levels, whereas the combined intervention of metformin + exercise led to a significant decrease in plasma copeptin levels. This finding suggests that the combination of exercise + metformin may have a potential effect in regulating copeptin levels. Enhörning et al. reported that high plasma copeptin levels in individuals with metabolic syndrome are associated with high fat intake and low physical activity [44]. These data suggest that lifestyle changes, particularly exercise, may be beneficial in managing metabolic diseases by reducing copeptin levels.

In this study, a significant increase in irisin levels was observed in the metformin + exercise group, although the most significant increase was seen in the exercise group. This finding suggests that both exercise and its combination with metformin may be effective in increasing irisin levels. On the other hand, lower irisin levels in the metformin + exercise group compared to those in the exercise group suggest that the combination of metformin and exercise has the potential to suppress the increase in irisin. This finding indicates that metformin, when used together with exercise, may weaken the enhancing effect of exercise on irisin levels. Some studies in the literature suggest that metformin may limit exercise-induced metabolic adaptations [45,46]. However, research indicates that both acute and chronic exercise can increase irisin levels [47,48,49,50,51,52]. A study by Taherzadeh et al. demonstrated that a combination of 6 weeks of aerobic exercise and Rosa canina seed extract supplementation in obese rats led to a significant increase in serum irisin levels compared to those in the control group [53]. Lu et al. showed that 8 weeks of swimming exercise significantly increased serum irisin levels in rats [54]. Irisin is a hormone that increases energy expenditure by converting white adipose tissue into brown adipose tissue in response to exercise [55,56], making such interventions potentially effective strategies for managing obesity.

Moreover, a positive, moderately significant correlation was found between irisin and adiponutrin (*r* = 0.50, *p* < 0.05), adropin (*r* = 0.52, *p* < 0.05), and copeptin (*r* = 0.59, *p* < 0.01), and a positive, weakly significant correlation was found between adropin and adiponutrin (*r* = 0.46, *p* < 0.05). A positive, strongly significant correlation was found between copeptin and adiponutrin (*r* = 0.73, *p* < 0.01). These findings suggest that these biomarkers may be involved in common physiological processes and may interact with each other.

This study has several limitations. First, exercise and metformin were not applied to non-obese rats, which may limit the generalizability of the findings to only obese rats. Second, only an aerobic exercise protocol was implemented, and the effects of other types of exercise were not considered. Third, only male Sprague–Dawley rats were used in the study, so the results can only be generalized to this gender and strain of rats. Finally, only blood samples were analyzed, and other biological samples (e.g., tissue samples) were not included.

## 5. Conclusions

In conclusion, the findings of this study demonstrate the effects of aerobic exercise and metformin on plasma uric acid, irisin, adiponectin, adropin, and copeptin levels in obesity. Specifically, changes in the levels of irisin, adropin, and copeptin emphasize the impact of exercise and metformin interventions on metabolic processes. Additionally, the attenuation of increased uric acid levels associated with obesity through exercise and metformin demonstrates the potential therapeutic effects of these interventions. However, the limited changes in adiponutrin levels indicate that further in-depth research is needed to fully understand the role of this molecule in metabolic responses.

## Figures and Tables

**Table 1:** Body mass (g) characteristics of the study groups. D: decapitation group, HC: healthy control group, S: sham group, OC: obese control group, M: metformin group, E: exercise group, ME: metformin + exercise group, SD: standard deviation, G: groups, wk: week; x^2^ (inter-wk): body mass differences between weeks were analyzed by the Friedman test; Z (intra-wk): body mass differences within weeks were analyzed by the Wilcoxon test; x^2^ (Wk × G): body mass differences between groups were analyzed by the Kruskal–Wallis test. The results are presented with lowercase letters (a, b, c), which indicate significant differences between groups and analyzed by the Mann–Whitney test. Uppercase letters (A, B, C) indicate differences resulting from the wk factor; *: *p* < 0.01, **: *p* < 0.001, ***: *p* < 0.001.

Groups	Baseline (g)	12th wk (g)	17th wk (g)	Inter-wk	Intra-wk	wk × G
	Mean ± SD	Mean ± SD	Mean ± SD			
D	231.4 ± 9.8	---	---	x^2^ = 54.000 *p* = 0.006 **	Baseline × 12th wk Z = 5.232, *p* = 0.0001 *** Baseline × 16th wk Z = 4.132, *p* = 0.0002 *** 12th wk × 16th wk Z = 2.245, *p* = 0.01 *	Baseline × G x^2^ = 0.930, *p* = 0.90 12th wk × G x^2^ = 17.003, *p* = 0.002 ** 16th wk × G x^2^ = 26.626, *p* = 0.0001 ***
HC	235.8 ± 11.0	370 ± 7.0 ^b^	430 ± 2.5 ^e^			
S	232.6 ± 9.1	649.6 ± 6.3 ^a^	685.7 ± 7.0 ^a^			
OC	233.0 ± 9.2	648.6 ± 5.4 ^a^	684.3 ± 3.3 ^a^			
E	237.8 ± 11.0	657.7 ± 5.7 ^a^	557.5 ± 5.8 ^b^			
M	233.3 ± 5.7	652.2 ± 7.7 ^a^	546.9 ± 5.0 ^c^			
ME	236.8 ± 8.6	656.0 ± 7.1 ^a^	507.7 ± 7.8 ^d^			
Wk Mean	234.7 ^C^	605.3 ^A^	568.1 ^B^			

## Data Availability

The raw data supporting the conclusions of this article will be made available by the authors on request.
